# Structure and Ecological Function of Fungal Endophytes from Stems of Different Mulberry Cultivars

**DOI:** 10.1007/s00284-023-03504-9

**Published:** 2023-11-06

**Authors:** Fangfang Peng, Xunlan Li, Zhaoxin Wei, Youjin Luo, Wu Wang, Guohui Han

**Affiliations:** Fruit Research Institute of Chongqing Academy of Agricultural Sciences, Chongqing, China

## Abstract

To explore the microbial community structure and ecological function of mulberry and their potential relationship with the resistance of mulberry, the community structure and function of endophytic fungi in 18 mulberry cultivars were analyzed and predicted by using high-throughput sequencing technology and the FUNGuild database. A total of 352 operational taxonomic units of fungi were observed at a 97% similarity level, representing six phyla of fungi, Fungi_unclassified, Ascomycota, Basidiomycota, Zygomycota, Rozellomycota, and Chytridiomycota. Fungi_unclassified was dominant, and Ascomycota was relatively dominant in all cultivars. At the genus level, *Ascomycota_unclassified* was dominant, and *Ampelomyces* was relatively dominant, with a richness in TAIWANCHANGGUOSANG 16.47–8975.69 times that in the other cultivars. Classified *Ascomycota_unclassified* was 4.75–296.65 times more common in NANYUANSIJI than in the other cultivars. Based on the FUNGuild analysis method, we successfully annotated six nutrient types, namely, pathotroph, pathotroph–saprotroph, pathotroph–saprotroph–symbiotroph, saprotroph, saprotroph–symbiotroph, and symbiotroph, among which saprophytic–symbiotic accounted for the largest proportion and was absolutely dominant in TWC. This research suggests that community composition differs among cultivars and that the diversity and richness of endophytic fungi in resistant cultivars are higher than those in susceptible cultivars. The ecological functions of cultivars with different resistances are quite different.

**Supplementary Information:**

The online version contains supplementary material available at 10.1007/s00284-023-03504-9.

## Introduction

Endophytes widely exist in plants and are an important part of plant microecosystems. In the long evolutionary process, they have established a special symbiotic relationship with parasitic plants, which can promote the growth of host plants, enhance their adaptability, enhance their tolerance to abiotic and biological threats, and promote the accumulation of secondary metabolites [1]. Studies have shown that endophytic fungi can regulate and control plant growth and assist the host plant in resisting severe environments by adjusting the levels of plant hormones and reactive oxygen species [2], such as gibberellin (GA) [3], salicylic acid (SA) [4], and jasmonic acid (JA) [5]. In addition, they can occupy the niche of pathogens and increase plant resistance to pathogens [6]. Different host plant genotypes, plant organs, developmental stages, health statuses, environments, etc., are important factors that affect endophytic diversity [7–10]. In turn, the community structure characteristics of plant endophytes form a dynamic indicator reflecting the growth environment and life characteristics of plants. Changes in endogenous communities have direct or indirect effects on the growth and survival of plants. Therefore, investigations into the distribution characteristics of plant endophytes can provide a theoretical reference for the further development and utilization of endophytic microbial resources.

Mulberry is a fruit-bearing tree species. As one of the representatives of the third generation of fruits, mulberry fruits are popular in the consumer market for their excellent nutritional value and health benefits [11]. With all the advantages mentioned, mulberry fruit effectively facilitates the transformation and upgrading of the mulberry industry. However, mulberry fruit sclerotiniosis still seriously threatens the development of the mulberry fruit industry. Such diseases mainly caused by four pathogens, namely, *Ciboria shiraiana*, *Ciboria carunculoides*, *Scleromitrula shiraiana,* and *Sclerotinia sclerotiorum* [12]. Among the diseases, mulberry fruit hypertrophic sclerotiniosis is the major one, with an incidence of up to 80% or more [13]. At present, the control of sclerotiniosis is mainly achieved by chemical pesticides [14]. Pesticide residues and environmental pollution bring great threats and risks to the green and safe production of mulberry [15]. Therefore, endophytes with antibacterial properties can be an effective choice for the biological control of sclerotiniosis [12].

Different mulberry cultivars have different levels of resistance to sclerotiniosis [16]. Is this difference related to differences in plant endophyte community structure and diversity? Wu et al. [17] analyzed the community structure and diversity of endophytes of four different resistant mulberry cultivars, and the results showed that numerous characteristics and the diversity of endophytes of resistant cultivars were significantly higher than those of susceptible cultivars. Endophytic bacterial populations of two citrus cultivars—the resistant satsuma mandarin and the susceptible Newhall navel orange—were analyzed through high-throughput sequencing, and the results showed that the differences in endophytic bacteria between citrus cultivars might be related to host resistance or susceptibility to citrus canker disease [18]. Endophytes of disease-resistant banana cultivars are more abundant and stable than those of disease-susceptible banana cultivars [19]. The composition of the root, stem, and leaf microbiotas of stripe rust-resistant wheat is higher than that of susceptible wheat [20]. An analysis of bacterial community composition in resistant and susceptible maize cultivars showed that the bacterial diversity index of resistant maize cultivars was higher than that of susceptible maize cultivars, and there was a significant difference in bacterial community composition among cultivars [21]. The above results show that there are some differences in the community structure and diversity of plant endophytes with different levels of resistance, which may be affected by the host genotype. Therefore, we wondered whether the community structure of endophytic fungi in different mulberry cultivars would be different and whether these differences are associated with disease resistance and ecological function. To answer these questions, we sequenced and analyzed the endophytic fungi of 18 mulberry cultivars from different ecological regions, aiming to obtain microbial information related to disease resistance and provide necessary basic information for evaluating the diversity and distribution of the endophytic fungi in mulberry and obtaining new microbial information.

High-throughput sequencing, known as “next-generation” sequencing technology, provides a new perspective in investigations on microbial diversity. Overcoming the limitations of noncultivability or low abundance in plate culture, this type of sequencing shows a great ability to quantify fungal diversity in environmental samples, thus representing an excellent technique for studying microbial diversity. However, a simple and consistent method is needed to classify a large number of sequences into one category or several categories of ecological significance. High-throughput sequencing technology was used in this study to investigate the diversity of endophytic fungi in different fruit mulberry cultivars, on the basis of which the fungal OTUs were classified into ecological guilds and analyzed by FUNGuild [22]. A preliminary discussion of the community structure and diversity of the endophytic fungi in mulberry fruit and the correlations between ecological functions and disease resistance provides theoretical references for the establishment of mulberry fruit microbial information systems, the ecological utilization of mulberry fruit microorganisms, and understanding mulberry fruit-microbe interactions.

## Materials and Methods

### Experimental Materials

Healthy branches from 18 cultivars of 5-year-old mulberry with different levels of resistance to sclerotiniosis were collected in this study. All samples were collected at the Chongqing Academy of Agricultural Sciences experimental farm. Healthy branches approximately 45.0 cm in length and 1.5–2.0 cm in diameter were collected in June (Table 1; Supplementary Fig. S1). The branches were washed in running tap water to remove surface debris and then cut into several stem segments (approximately 3–4 cm). Surface-sterilized stem segments from each cultivar were randomly selected, and the stem segments were completely immersed in 75% ethanol for 30 s, washed with sterile water, soaked with 3% H_2_O_2_ for 3 min, and then rinsed with sterile water 3–4 times. The last time, sterile water was spread on PDA medium to test the sterilization effect. Sterile stem segments were used for DNA extraction.

**Table 1:** Origin and resistance characteristics of cultivars used in this study

No	Cultivar	Code	Origin	Resistance level	No	Cultivar	Code	Origin	Resistance level
1	DASHI	DS	GUANG DONG	Susceptible	10	DABAIE	DBE	HEBEI	Highly resistant
2	YUMEN YIHAO	YM	SICHUAN	Moderately resistant	11	LONGSANG	LS	SICHUAN	Resistant
3	YUANKUN YIHAO	YK	SICHUAN	Resistant	12	TU13	T13	XINJIANG	Moderately resistant
4	NANYUAN SIJI	NYSJ	TAIWAN	Highly resistant	13	TU6	T6	XINJIANG	Susceptible
5	DECHANG YIHAO	DC	SICHUAN	Moderately resistant	14	KA3	K3	XINJIANG	Moderately resistant
6	TAIWANCHANG GUOSANG	TWC	TAIWAN	Immune	15	A6	A6	XINJIANG	Susceptible
7	JIZHUA SANG	JZS	ZHEJIANG	Resistant	16	A7	A7	XINJIANG	Moderately resistant
8	BAIYUWANG	BYW	SHANDONG	Highly resistant	17	HONGGUO ERHAO	HG	SHANXI	Susceptible
9	ZHENZHU BAI	ZZB	SHANDONG	Susceptible	18	LVSHENZI	LSZ	SHANDONG	Susceptible

The degree of resistance is divided into five levels: immune, highly resistant, resistant, moderately resistant and susceptible [16]

### Genomic DNA Extraction, PCR Amplification and Detection of Endophytes in Stems

The sterilized stem segments were thoroughly ground with liquid nitrogen and then transferred into sterilized EP tubes. Total DNA was extracted by using a DNA extraction kit according to the manufacturer’s instructions. The concentration and purity of the extracted genomic DNA were detected by 1% agarose gel electrophoresis. According to the sequencing area, specific primers with barcodes were synthesized for PCR amplification. An AxyPrepDNA gel recovery kit (AXYGEN company) was used to cut the gel to recover the PCR products, which were eluted with Tris_HCl and detected by 2% agarose electrophoresis.

PCR was carried out in a 20 μl volume with the following reagents: 5× FastPfu Buffer 5 μl, 2.5 mM dNTPs 2.5 μl, forward primer (5 μM) 1 μl, reverse primer (5 μM) 1 μl, FastPfu polymerase 0.5 μl, BSA 0.25 μl, template DNA 10 ng, and ddH2O added to 20 μl. Thermal cycling consisted of one cycle of 95 ℃ for 3 min, followed by 32 cycles of 94 ℃ for 30 s, 55 ℃ for 30 s, and 72 ℃ for 45 s, and a final extension at 72 ℃ for 5 min. The ITS1 region of the fungal rRNA gene was amplified using the primers ITS1-F (5′-CTTGGTCATTTAGAGGAAGTAA-3′) and ITS2-R (5′-GCTGCGTTCTTCATCGATGC-3′). With reference to the preliminary quantit-ST Blue Fluorescence Quantitative System (Promega Company), the samples were homogenized uniformly.

### Illumina PE250 Library Construction and Sequencing

Library construction was performed using a TruSeq^®^ DNA PCR-Free Sample Preparation Kit. The constructed libraries were quantified by a Qubit instrument and qPCR. Thereafter, amplicons were pooled in equal amounts, and paired-end 2× 250 bp sequencing was performed with the NovaSeq-PE250 approach on the Illumina MiSeq platform.

### Data Processing and Analysis

Sequence optimization and statistical analysis were performed by using Trimmomatic, Flash, Usearch, QIIME, and a custom Perl script from Shanghai Yuanxin Bio. The effective sequences were clustered into OTUs according to a similarity of 97%. Species taxonomic analyses were carried out on the QIIME platform and with RDP Classifier by using the OTU representative sequences with a similarity level of 97% [23], and then the microbial diversity index was calculated. The community heatmap was constructed and PCoA was performed in the R environment [24, 25]. Fungal functional type analysis was performed by the FUNGuild tool [22, 26].

## Results

### Sequencing Data Analysis

According to the PCR product inspection of 18 samples, the quality of YM and LSZ was grade C (No. 2 and No. 18), and owing to the extremely weak bands, subsequent tests could not be carried out. The results of the remaining 16 samples were graded as A or B, which enabled the next step to proceed (Supplementary Fig. S2). After the 16 qualified samples were filtered through quality control and the chimeras were removed, we obtained a total of 1,150,966 endophytic fungal effective tags with a base number of 328,818,112 bp and an average length of 285.69 bp. A total of 352 OTUs were obtained by performing clustering at a 97% similarity level. The rarefaction dilution curve showed that each sample reaches stability when the sequencing arrives at a certain level, indicating that the amount of sequencing data was reasonable and that the sample sequencing depth was good. In the specaccum species accumulation box plot, as the sample size increases, the box plot gradually becomes flat, indicating no significant increase in the fungal species in the environment with the increase in sample number, which means that the sampling is adequate and data analysis can be carried out (Supplementary Fig. S3).

### Endophytic Diversity Analysis

The microbial diversity index is an effective metric for assessing microbial diversity. The alpha diversity index is usually assessed by four indicators: Ace, Chao, Shannon, and Simpson [27]. The Ace index and Chao index are used to assess community richness: a larger value means a higher abundance. The Shannon index and Simpson index are used to assess community diversity: a larger Shannon index means higher community diversity, and a larger Simpson index means lower community diversity. In this study, three indices, namely, the Chao index, Shannon index, and Simpson index, were adopted. All the sequencing depth coverage index of the samples exceeded 0.9999 (Table 2), indicating the completeness of the sequencing data and the low probability of undetected sequences of fungi of each sample. According to the Shannon and Simpson diversity index, the cultivars with significantly higher endophytic diversity were NYSJ, TWC, DBE, YK, and JZS. According to the Chao index, the cultivars with higher endophytic abundance were TWC, NYSJ, YK, DBE, and JZS. It is shown that there are certain differences among the 16 cultivars in terms of the diversity and richness of the endophytic community, and the above 5 cultivars have advantages in diversity and richness.

**Table 2:** Diversity index of endophytic fungal communities in different cultivars

Cultivar	Coverage	Shannon	Simpson	Chao
A6	0.99999	0.29	0.93	39
A7	1	0.57	0.84	51
BYW	1	0.36	0.88	34
DBE	0.99997	1.26	0.6	54
DC	1	0.81	0.73	49
DS	0.99997	0.9	0.71	37
HG	1	0.87	0.73	41
JZS	0.99996	1.06	0.69	53
K3	0.99999	0.85	0.53	13
LS	1	0.79	0.77	53
NYSJ	1	2.78	0.18	70
T13	1	0.97	0.69	35
T6	0.99999	0.84	0.75	46
TWC	1	2.08	0.27	81
YK	0.99995	1.16	0.67	59
ZZB	0.99999	0.7	0.77	38

**Fig. 1:**
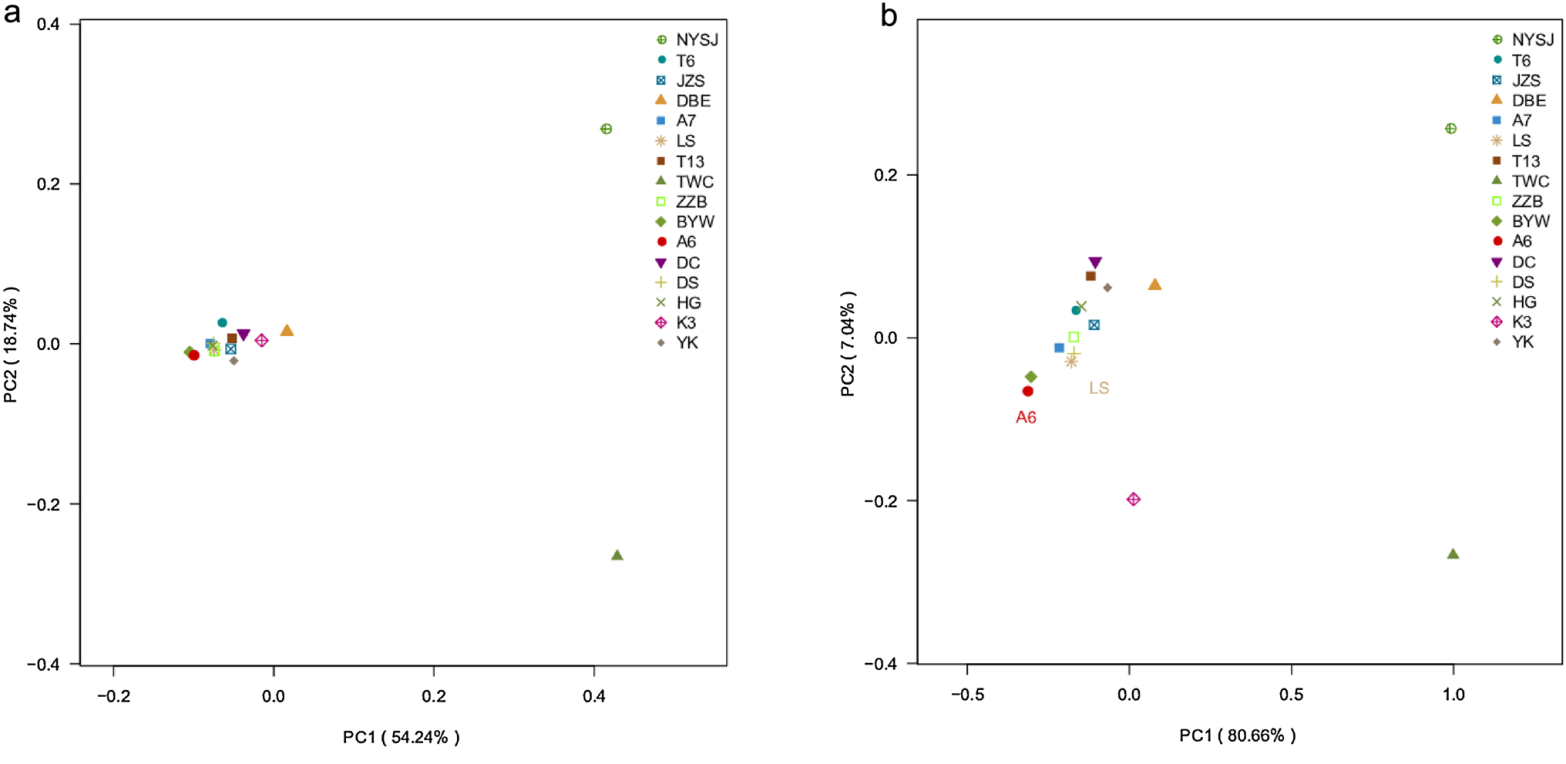
PCoA of endophytic fungal communities in different cultivars. **a** PCoA based on the Bray‒Curtis algorithm. **b** PCoA based on the weighted UniFrac algorithm. The PCoA results based on the Bray‒Curtis algorithm and weighted UniFrac algorithm showed that there were obvious differences between TWC, NYSJ, and other cultivars

In this study, principal coordinate analysis (PCoA) was used to analyze the beta diversity of 16 cultivars. Two weighted analysis methods, bray_curtis and weighted_unifrac, were used to address the problem concerning the existence and abundance of species in each sample. The Bray‒Curtis algorithm focuses more on the differences in the OTU structures (including composition and abundance) of the microbial communities, while UniFrac attaches more importance to the evolutionary classification of the communities. PC1 and PC2 explained 54.24% and 18.74% of the variation in endophytic fungal communities, respectively, and together, they explained 73.98% of the variation in community structure (Fig. 1a). At the OTU level, TWC and NYSJ were relatively dispersed, with relatively long distances from the other 14 cultivars of concentrated distribution, which indicates that there are significant differences between TWC and NYSJ and the other 14 cultivars in terms of the composition and abundance of endophytes. PC1 and PC2 explained 80.66% and 7.04% of the variation in endophytic communities, respectively, and together, they explained 87.7% of the variation in community structure (Fig. 1b). From the perspective of the differences in the evolutionary classification of the communities, TWC, K3, and NYSJ showed a dispersed distribution, with relatively long distances from the other 13 cultivars of concentrated distribution, which indicates the large evolutionary differences between TWC, NYSJ, and K3 and the other 13 cultivars in terms of endophytic communities.

### Community Structure of Endophytes

**Fig. 2:**
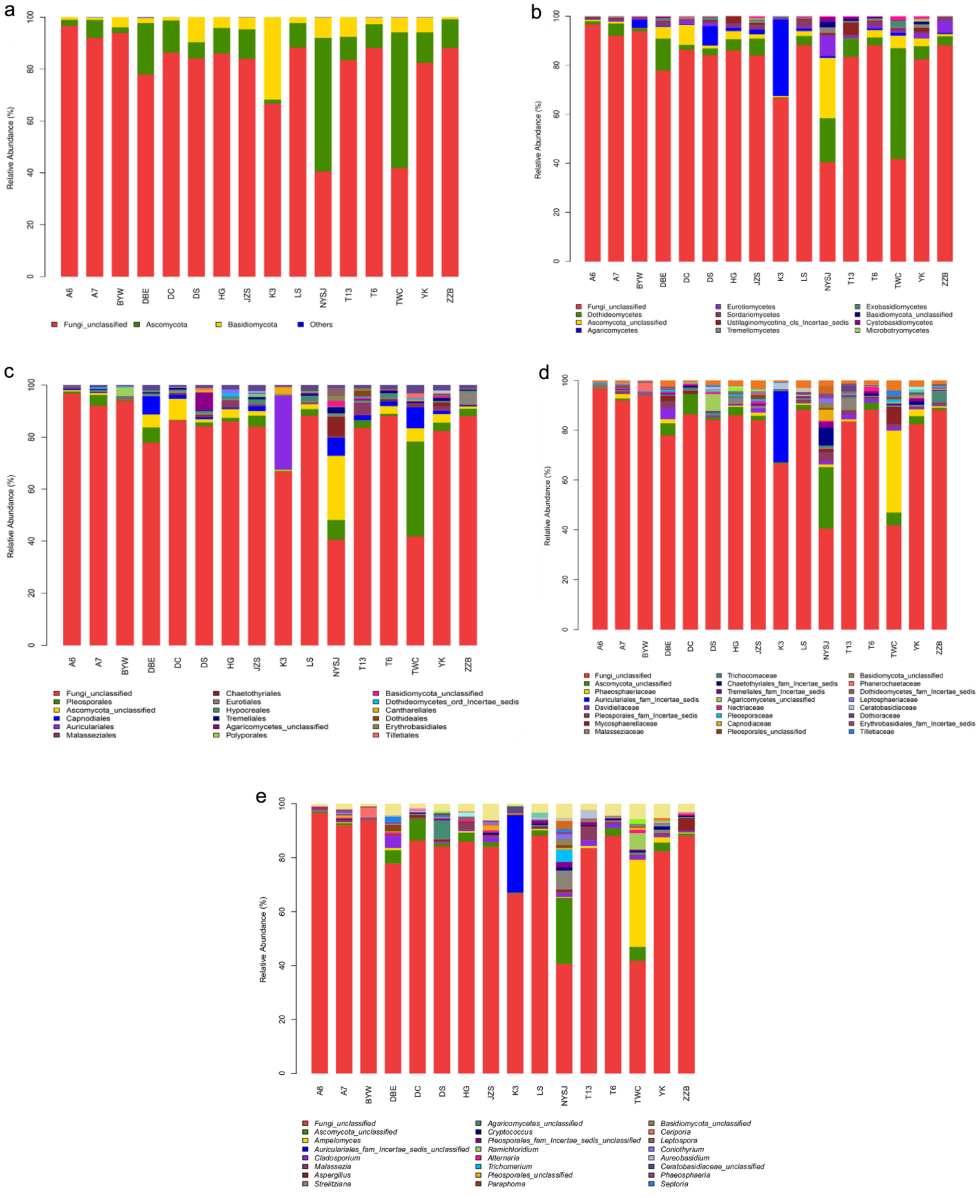
The main endophytic fungal microbiomes at different taxonomic levels in different mulberry cultivars. **a** Top 3 microbiomes at the phylum level; **b** top 12 microbiomes at the class level; **c** top 18 microbiomes at the order level; **d** top 24 microbiomes at the family level; **e** top 24 microbiomes at the genus level

An analysis was conducted on the endophytic community structures of 16 cultivars at the levels of genus, family, order, class, and phylum. As shown in Fig. 2, 6 phyla of fungi, Fungi_unclassified (80.02%), Ascomycota (14.17%), Basidiomycota (5.75%), Zygomycota (0.04%), and Rozellomycota (0.007%) and Chytridiomycota (0.01%) at low abundance, were deteted. Fungi_unclassified was the dominant phylum, and Ascomycota was the relatively dominant phylum, including 9 classes, 33 orders, 56 families, and 109 genera. Basidiomycota includes 8 classes, 20 orders, 29 families, and 43 genera. Zygomycota and Rozellomycota are relatively simple, with 1 class, 1 order, 1 family, and 1 genus each. At the class level, Dothideomycetes (7.70%) was the dominant class, and Ascomycota_unclassified (3.77%) was the relatively dominant class. The classes accounting for more than 1.0% were Agaricomycetes (3.20%), Eurotiomycetes (1.48%), and Sordariomycetes (1.0%), while those accounting for 0.3%–1.0% were Ustilaginomycotina_cls_Incertae_sedis (0.83%), Tremellomycetes (0.6%), and Exobasidiomycetes (0.38%). At the order level, Pleosporales (4.71%) was dominant, and Ascomycota_unclassified (3.77%) was relatively dominant. The remaining orders accounting for over 1.0% were Capnodiales (2.07%) and Auriculariales (1.80%), while those accounting for 0.3%–1.0% were Malasseziales (0.83%), Chaetothyriales (0.74%), Eurotiales (0.74%), Hypocreales (0.67%), Polyporales (0.35%), Tremelales (0.56%), and Agaricomycetes_unclassified (0.52%). At the family level, Ascomycota_unclassified (3.77%) was the dominant family, while Phaeosphaeriaceae (2.77%) was a relatively dominant family. The one family accounting for more than 1.0% was Auriculariales_fam_Incertae_sedis (1.80%), and 12 families accounted for 0.3%–1.0%, including Davidiellaceae (0.95%). At the genus level, *Ascomycota_unclassified* (3.77%) was dominant, and *Ampelomyces* was relatively dominant (2.39%). The genus accounting for over 1.0% was the genus *Auriculariales_fam_Incertae_sedis*_*unclassified* (1.78%), and those accounting for 0.3%–1.0% included 11 genera, such as *Cladosporium* (0.95%).

### Differences in Endophytic Community Structure Among Cultivars

**Fig. 3:**
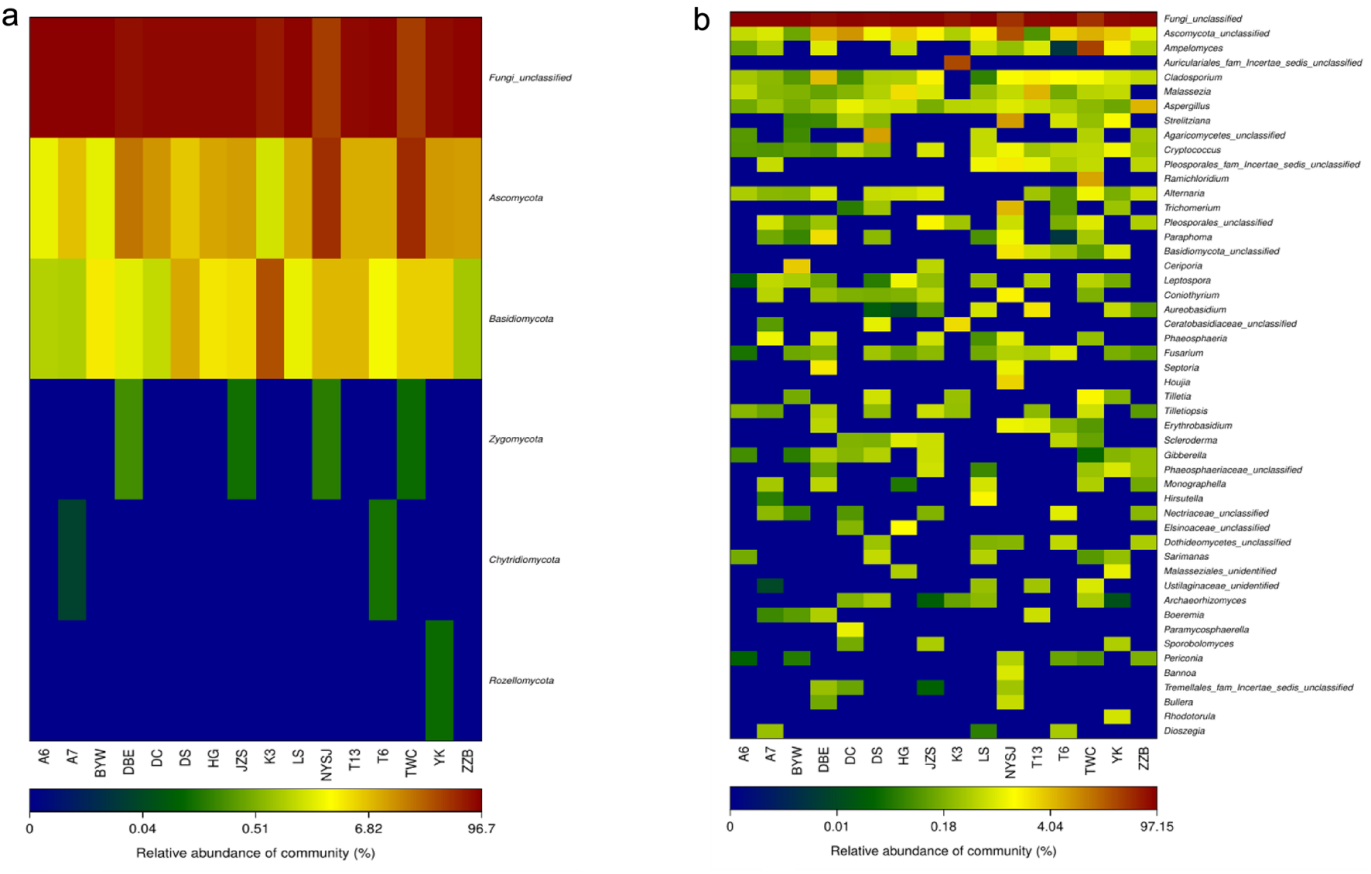
Heatmap of endophytic fungal community structures at the phylum and genus levels. The top 50 phyla (**a**) and genera (**b**) with the highest abundance in each group were selected. The different color gradients reflect the similarities and differences in the community compositions of different cultivars at different taxonomic levels

To study the similarities and differences in community structure among different cultivars, heatmaps at the phylum level and genus level were constructed. Heatmaps can intuitively display the values of the data with a defined color depth [24]. As shown in Fig. 3a, at the taxonomic level of phylum, Fungi_unclassified accounts for 40.53%–96.70% of each sample, which is a relatively large amount, with Ascomycota accounting for over 1% of each sample. Therein, TWC, NYSJ, and DBE rank as the top three, with values of 52.40%, 51.47%, and 19.83%, respectively; K3 accounts for 1.59%, the smallest proportion. Basidiomycota accounts for less than 1% in each sample, only exceeding ZZB. The proportions of the remaining samples are in the range of 1.05%–31.72%. Zygomycota was found only in four cultivars, namely, NYSJ, JZS, DBE, and TWC, accounting for 0.17%, 0.12%, 0.24%, and 0.11%, respectively. The chytrid phylum was found in A7 (0.03%) and T6 (0.13%). Rozellomycota was found in YK (0.11%). A total of 141 genera were detected in the 16 cultivars, where the top 50 were analyzed by a heatmap at the taxonomic level (Fig. 3b). Overall, the community structures of the 16 cultivars were quite different, sharing few common genera. In addition to *Fungi_unclassified*, *Ascomycota_unclassified* and *Aspergillus* were also common genera among the 16 samples. At the genus level, TWC had the highest fungal diversity, NYSJ and DBE had the second highest diversity. Some cultivars contained unique genera: *Ramichloridium* was found only in TWC; *Rhodotorula* was found in YK; *Bannoa* and *Houjia* were found in NYSJ; and *Auriculariales_fam_Incertae_sedis*_*unclassified* was found in K3, with a relatively high abundance. Generally, *Ampelomyces* and *Ascomycota_unclassified* showed significant differences in abundance among cultivars. The abundance of *Ampelomyces* in TWC was 16.47–8975.69 times that in the other cultivars. The abundance of *Ascomycota_unclassified* in NYSJ was 4.75–296.65 times that in the other cultivars. The above results show that there are some differences in the fungal community structures and diversities of the 16 cultivars, with some cultivars showing distinct communities.

### Functional Prediction of Endophytes

**Fig. 4:**
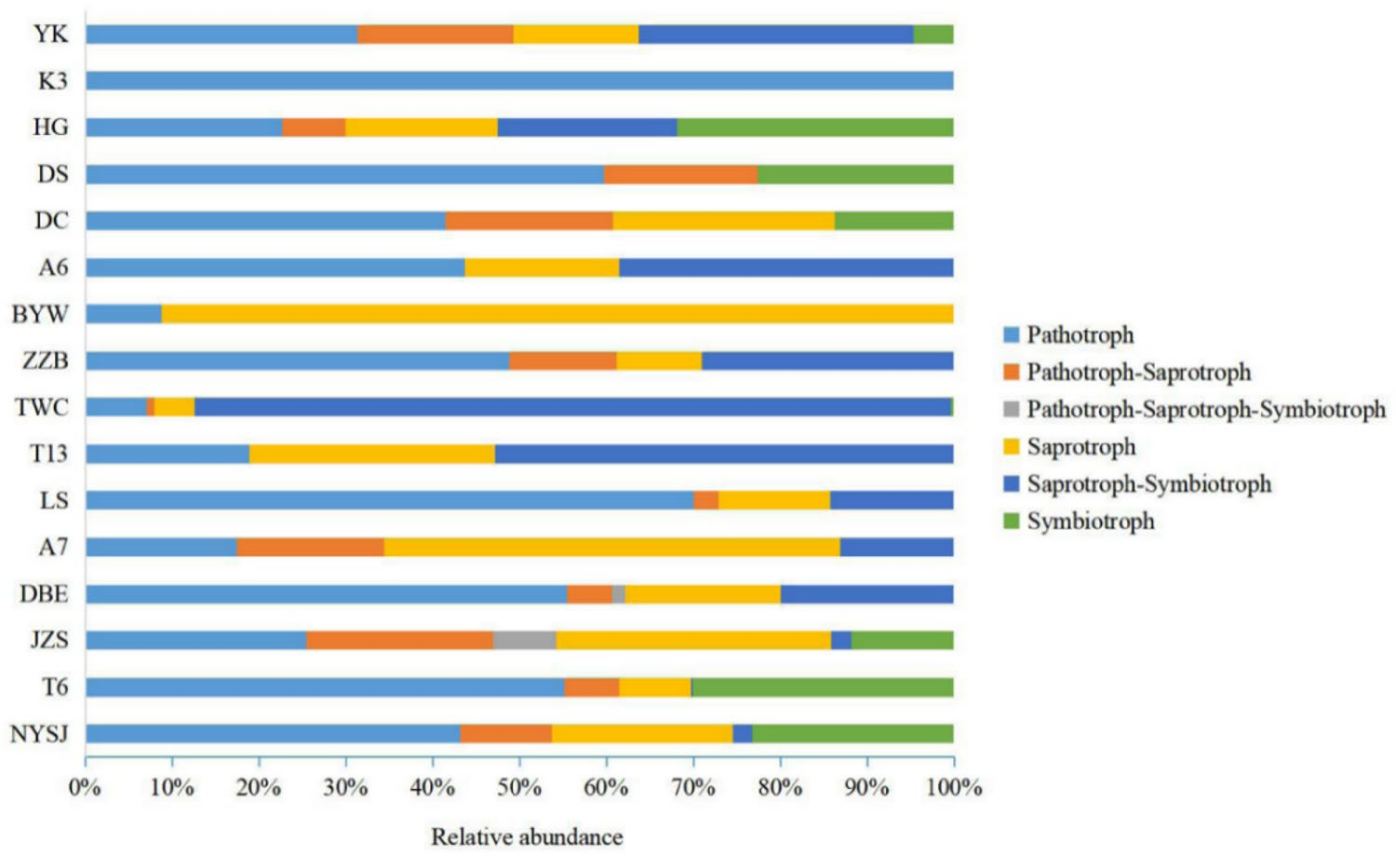
Trophic composition of endophytic fungal communities in different cultivars. Using FUNGuild to predict the function of endophytic fungi, we found 6 nutritional modes (pathotroph, pathotroph-saprotroph, pathotroph-saprotroph-symbiotroph, saprotroph, saprotroph-symbiotroph, and symbiotroph)

FUNGuild can link the fungal gene sequence information obtained by methods such as high-throughput sequencing/cloning libraries with the ecological function of fungi. Based on this, FUNGuild was used in this study to predict the functions of endophytes in 16 cultivars and allocate them to different trophic types and colocation groups (Fig. 4). Nutritional function information was successfully annotated into 6 categories, namely, pathotroph, pathotroph–saprotroph, pathotroph–saprotroph–symbiotroph, saprotroph, saprotroph–symbiotroph, and symbiotroph. The nutrient abundances and structures of the cultivar were different. In general, the nutritional type accounting for the largest proportion was saprotroph–symbiotroph, with an absolute advantage in TWC. The second largest nutritional type was pathotrophs, which were found in all samples. The third most common nutritional type was saprotrophs, which had an absolute advantage in BYW, with only a small number in TWC.

**Fig. 5:**
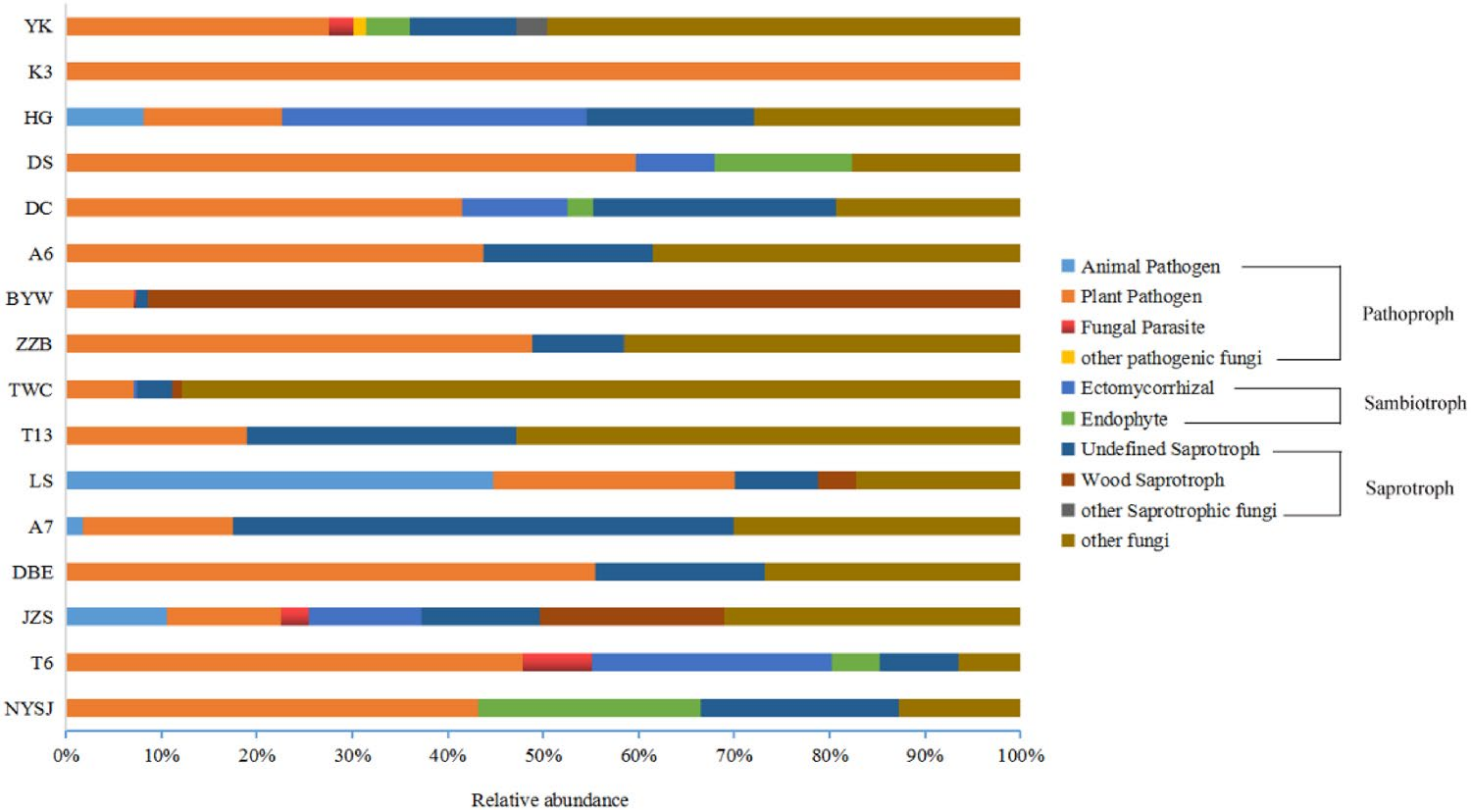
Variations in the composition of endophytic fungal functional communities in different cultivars. The top 9 with the highest percentage were selected, and the rest were assigned to “others fungi”

As shown in Fig. 5, we predicted 10 ecological colocation groups in the 16 cultivars (at the genus level), including animal pathogen, plant pathogen, fungal parasite, ectomycorrhizal, other pathogenic fungus, endophyte, wood saprotroph and undefined saprotroph, as well as two types of mixotrophic fungi. The fungal types by pathotroph include animal pathogen, plant pathogen, fungal parasite, and other pathogenic fungi. The relative abundance of animal pathogens in LS (44.75%) was significantly higher than that in other cultivars, and the taxonomic unit was *Hirsutella*. The relative abundance of plant pathogens in K3 (100%) was significantly higher than that in the other cultivars, and the taxonomic unit was *Tilletia*. Fungal parasites were found only in T6 (7.29%), JZS (2.94%), BYW (0.20%), and YK (2.53%), with relatively low abundances. The fungal types of symbiotrophs included ectomycorrhizae and endophytes. The relative abundance of ectomycorrhizae in HG (31.89%) was significantly higher than those in other cultivars, and T6 (25.05%), JZS (11.81%), and DC (11%) came in second, all falling into the taxonomic unit of *Scleroderma*. The relative abundance of endophytes in NYSJ (23.27%) was significantly higher than that in other cultivars, and the taxonomic unit was *Trichomerium*. The fungal types of saprotrophs include undefined saprotrophs, wood saprotrophs, and other saprotrophic fungi. The relative abundance of undefined saprotrophs in A7 was significantly higher than that in the other cultivars, with *Phaeosphaeria* being the absolutely dominant genus (83.88%). Wood saprotrophs were found only in BYW, JZS, TWC, and LS, with an absolute dominance in BYW owing to their relative abundance (91.47%), and the taxonomic unit was *Ceriporia*. The relative abundance of other saprophytic fungi was very low, and these fungi were found only in YK and ZZB. The relative abundance of other fungi in TWC reached 87.90%, a value significantly higher than those in other cultivars, with *Ampelomyces* being the absolutely dominant genus (98.69%). Cluster analysis was carried out for the purpose of visualizing the differences in the ecological functions of the fungi among the cultivars (Supplementary Fig. S4). By the reference line, the 16 cultivars were divided into 5 categories: TWC, NYSJ, and BYW fell into one category, DBE and YK were clustered into another category, and the remaining 11 cultivars were clustered into another category. This indicates that there are large differences between the ecological function groups of TWC, NYSJ, and BYW and those of other cultivars.

## Discussion

Rich in resources, endophytic fungi are of significant research value in fields such as agriculture, medicine, industry [28]. One or more endophytic fungi are found in most plants [29]. At present, there are no reports available on the diversity of endophytic fungi in mulberry. Several studies have been conducted for detection of their endophytic bacteria, but with few cultivars of mulberry. This study involves investigations into the diversities of endophytes of 18 mulberry cultivars. The results show that there are certain differences concerning the diversities of endophytes in different hosts of the same cultivars. Therein, the PCR results of 2 cultivars showed small fungal contents, preventing subsequent analysis. According to the Shannon index of the remaining 16 cultivars, the cultivars with a value greater than 1 were NYSJ, TWC, DBE, YK, and JZS, and the rest had values were between 0.29 and 0.90. Studies show that an increase in the diversity of fungal communities enhances the positive colony effect of microorganisms and improves the comprehensive resistance of host plants against pathogens, thereby helping to promote fruit yield and quality [30]. According to relevant reports [23, 24, 31] and observations on the incidence of sclerotiniosis at the mulberry fruit base of the Chongqing Academy of Agricultural Sciences, TWC is an immune cultivar against sclerotiniosis, and JZS, DBE, NYSJ, and YK display good disease resistance, indicating that a higher microbial community diversity index means higher resistance, which is in line with the results of Allison [32] and Wu et al. [17].

Plant endophytic fungi mainly include Ascomycota, Basidiomycota, and Zygomycota [33]. In this study, the endophytic fungi in the mulberry stems included 5 phyla in addition to Fungi_unclassified, namely, Ascomycota, Basidiomycota, Zygomycota, and a very small amount of Rozellomycota and Chytridomycota. Ascomycota is the dominant phylum, which is similar to the case of mulberry fruit [34]. The study indicates that there are significant differences among the community structures of different cultivars. Ascomycota and Basidiomycota are common phyla, and Zygomycota was found only in four typical cultivars, namely, NYSJ, JZS, DBE, and TWC. A small number of Chytridiomycota were found in A7 (0.03%) and T6 (0.13%), the two cultivars collected in Xinjiang. Rozellomycota was found in YK. It is speculated that these differences may be closely related to the mulberry cultivar or resistance. As shown in the heatmap graph, specific genera or dominant genera with significantly high abundances exist in several typical mulberry cultivars. The most noticeable genus was *Ampelomyces*, of which the abundance in TWC was 16.47–8975.69 times that in the other cultivars. It is considered to be a biocontrol bacterium of epiparasitism. The secretion of antibacterial quinone compounds [35] directly affects the health of plants during the interaction between microorganisms and plants, thus improving plant disease resistance [36]. Some strains were later registered as commercial fungicide products after being used as biocontrol bacteria for the prevention and control of powdery mildew in important crops such as grapes and cucumbers [37]. Importantly, they are not just the host strains of powdery mildew. They are also speculated to be parasitic on other pathogenic fungi, including *Botrytis cinerea*, *Alternaria solani*, *Colletotrichum coccodes,* and *Cladosporium cucumerinum* [38]. Such findings provide a new approach for the biological control of mulberry fruit sclerotiniosis. In addition, a large number of *Ascomycota_unclassified* existing in NYSJ are likely to fall within a new species that is speculated to be capable of playing an important role in biological control.

Although sequencing technology allows researchers to obtain large microorganism resources, many of the microorganism cultivars may play only a minor role in ecological functions [39]. In this study, relevant functional information for 77.00%–98.74% of OTUs was not obtained, which is speculated to be because access to functional prediction information is limited owing to the incomplete information in the fungal function prediction database [22] or because the current investigations into mulberry endophytic fungi are still too scarce, which means that microbial resources and ecological functions need to be further developed. The study indicates that there are certain differences in the nutritional types and ecological flora among the cultivars. Overall, saprotroph–symbiotroph, pathotroph, and saprotroph were the main types. According to the clustering results, the typical species are TWC, NYSJ, BYW, DBE, and YK, which is in line with the results of the endophytic diversity analysis. Therefore, combining the results of endophytic fungal diversity and ecological function prediction, it can be inferred that the endophytic fungal diversity and richness of the cultivars with strong disease resistance are higher, and the ecological function is significantly different from that of the susceptible cultivars. The following points need to be emphasized: the abundance of plant pathogens in TWC was the lowest, and other fungi were highly abundant. *Ampelomyces* was the absolutely dominant genus (98.69%) among the other fungi. According to the analysis above, the high resistance of TWC to sclerotiniosis may be correlated with *Ampelomyces*. BYW has a low abundance of plant pathogens and an absolute predominance of wood saprotrophs. As the dominant genus that grows in BYW only, *Ceriporia* (91.47%) has many functions in agriculture, and it can improve the utilization efficiency of soil nutrients by crops [40, 41]. Notably, *Ceriporia* can also synthesize a series of secondary metabolites and has gained attention as a microbial source of biocatalysts for the biotransformation of natural terpenoid products [42]. The in vitro antifungal activities of terpenoids against a variety of plant pathogenic fungi were evaluated by the mycelial growth rate method, and the results showed that terpenoids exhibited broad-spectrum antifungal activity [43]; thus, the fungi can be considered potential biocontrol fungi [44]. Although the relative abundance of pathotrophs in LS is high, the dominant fungus (*Hirsutella*) is a parasitic fungus that has a good biological control effect on plant pathogenic nematodes such as mites, stem nematodes, root-knot nematodes, and some lepidopteran insects [45].

## Conclusion

In this study, high-throughput sequencing technology was applied to obtain information on the endophyte communities in the stems of different mulberry cultivars. By comparative analyses, we found that there are certain differences in terms of the diverse structures of endophyte communities of different cultivars and that the higher the microbial diversity is, the stronger the plant resistance is. It was also found that the resistant endophytic fungi demonstrate particularity in quantity or strains, which may be of certain value in investigations into plant resistance. Furthermore, cluster analysis also showed differences between mulberries with high resistance and other species in terms of ecological function. The research results can provide theoretical references for the construction of mulberry microbial information systems, the screening of antagonistic strains against sclerotinia, the ecological utilization of mulberry microorganisms and the interactions between mulberry and microbes.

### Supplementary Information

Below is the link to the electronic supplementary material.

Supplementary file1 (DOCX 407 KB)
