# Supplementary material for: Structure and Ecological Function of Fungal Endophytes from Stems of Different Mulberry Cultivars
Source: Curr Microbiol. 2023 Nov 6;80(12):401. doi: 10.1007/s00284-023-03504-9 (PMC10628033; doi:10.1007/s00284-023-03504-9)
Supplement: Supplementary file 1 — Supplementary file1 (DOCX 407 KB) [file 284_2023_3504_MOESM1_ESM.docx]

### *Supplementary Information*

**Structure and Ecological Function of Fungal Endophytes from Stem of Different Mulberry Cultivars**

Fangfang Peng^1#^, Xunlan Li^1#^, Zhaoxin Wei^1^ , Youjin Luo^1^, Wu Wang^1^, Guohui Han^1^*

^1^ Fruit Research Institute of Chongqing Agriculture Science Academy,Chongqing, China

# Fangfang Peng and Xunlan Li contributed equally to this work .

*For correspondence:Guohui Han, E-mail: [hghui2007@126.com](mailto:hghui2007@126.com).

***Supplementary figure***


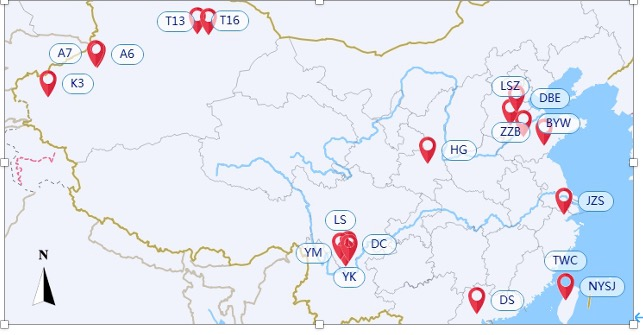


Supplementary Fig. S1 Cultivar origin and distribution. Each red mark on this map represents the location where a mulberry germplasm resource was collected. The resources were eventually preserved at the mulberry base of Chongqing Academy of Agricultural Sciences


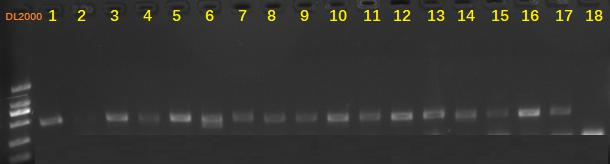


Supplementary Fig. S2 PCR map of 18 mulberry cultivars. The yellow numbers in the figure represent the numbers of the cultivars. Among them, the quality inspection results for No. 2 and No. 18 did not reach the test standards, so subsequent tests could not be carried out


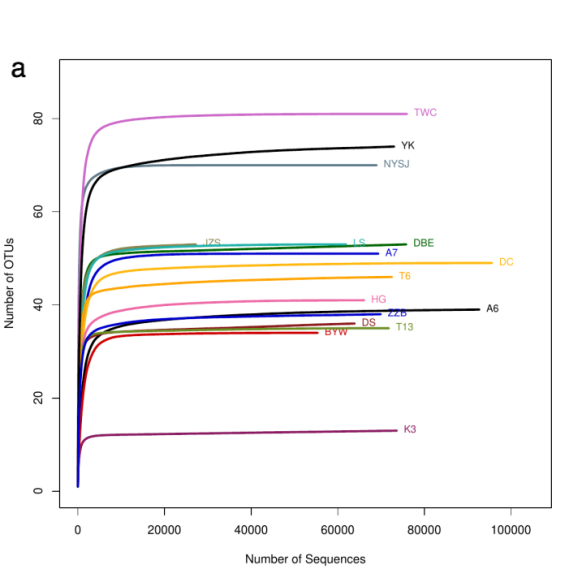

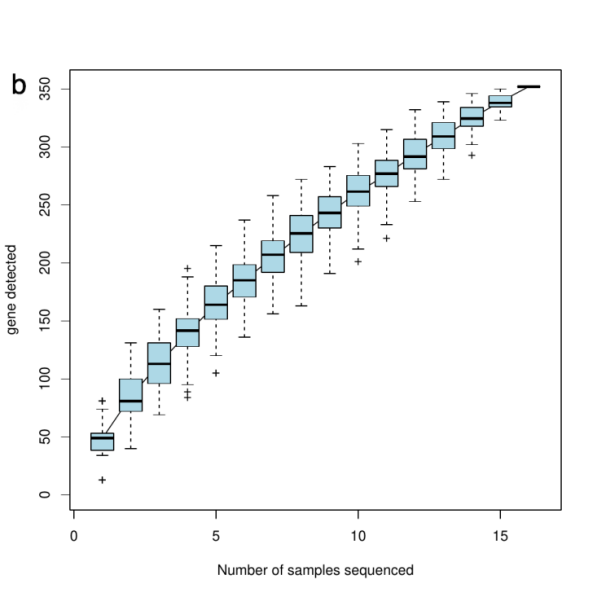


Supplementary Fig. S3 Rarefaction curve (a) and species accumulation curve (b). When the rarefaction curve tends to be flatten, it indicates that the numbers of sequencing data points are reasonable; when the species accumulation curve tends to flatten, it indicates that the numbers of samples are sufficient and that the data can be analyzed





Supplementary Fig. S4 Functional prediction cluster analysis of endophytic fungal communities in different cultivars. Based on the functional prediction data, 16 cultivars were grouped into 5 categories by the dividing line
